# Beyond greenness: multidimensional urban nature profiles and arteriosclerotic cardiovascular risk

**DOI:** 10.1016/j.envint.2025.110033

**Published:** 2025-12-30

**Authors:** Pablo Knobel, Aditi Singhvi, Helena Krasnov, Elena Colicino, Itai Kloog, Rachel Litke, Kevin Lane, Alex Federman, Charles Mobbs, Maayan Yitshak Sade

**Affiliations:** a Department of Environmental Medicine, Icahn School of Medicine at Mount Sinai, New York, New York, the United States of America; b Nash Family Department of Neuroscience, Friedman Brain Institute, Icahn School of Medicine at Mount Sinai, New York, NY, the United States of America; c Department of Environmental Health, Boston University School of Public Health, Boston, MA, the United States of America; d Division of General Internal Medicine, Icahn School of Medicine, New York, NY, the United States of America

## Abstract

Urban nature, including green and blue spaces, vegetation cover, and biodiversity, has been linked to improved cardiometabolic health. However, most exposure metrics oversimplify complex environmental conditions, limiting their relevance for public health and equity. We developed a multidimensional classification of urban nature using eight high-resolution indicators within 300-meter residential buffers for 36,830 New York City residents aged ≥ 55 years. Using k-means clustering, we identified five distinct exposure profiles based on their mean feature: Low Green, Street Trees, High Cover, Park Access, and Waterfront. We estimated associations between these profiles and incident atherosclerotic cardiovascular disease (ASCVD) from 2013 to 2022 using Cox proportional hazards models with competing risks, and evaluated effect modification by sex, race/ethnicity, and neighborhood-level poverty and racial composition. Over a median follow-up of 3.8 years, 28.7 % of participants experienced an ASCVD event. Compared with the Low-green profile, ASCVD risk was lower for Waterfront (HR = 0.88; 95 % CI: 0.81–0.95), Park Access (0.89; 0.84–0.94), Street Trees (0.92; 0.87–0.98), and High Cover (0.95; 0.88–1.02) profiles in fully adjusted Cox models. Significant interaction term indicated that the association between the Park Access profile and ASCVD risk differed by sex, with stronger protective associations observed for males (interaction p-value: 0.04). Similarly, Street Trees were more protective in areas with higher percentages of non-Hispanic Black residents (interaction p-value: 0.02). These results underscore the value of multidimensional urban nature metrics for understanding and promoting cardiovascular health equity in dense cities.

## 1. Introduction

Atherosclerotic cardiovascular disease (ASCVD) remains a leading cause of death in the United States, with age-adjusted mortality rates rising from 7.7 % in 2018 to 8.5 % in 2020 (Shahid et al., 1999). The global population ages and ASCVD is projected to rise from 17 % in 2024 to 33 % by 2054 (United Nations, 2024). Consequently, the burden of ASCVD and other age-related cardiometabolic diseases is expected to intensify (Yusuf et al., 2001; Yusuf et al., 2001). Risk patterns vary significantly by age, race, sex, geography, and environmental context (Rodgers et al., 2019; Gebreab et al., 2015; Huang et al., 2024). Emerging evidence positions environmental exposures among the key drivers of ASCVD risk, in some cases even surpassing clinical risk factors (Brown et al., 2024; Stephens et al., 2019; Twohig-Bennett and Jones, 2018). Collectively referred to as urban nature; features such as parks, street trees, riverbanks, and habitat diversity are associated with better cardiometabolic health (Fong et al., 2018; James et al., 2015; Kondo et al., 2018; Gascon et al., 2018; Nwana et al., 2024).

Different frameworks (Pretty, 2004; Nieuwenhuijsen et al., 2017; Markevych et al., 2017) underscore the complexity of how urban nature influences health. It is not only the mere presence of vegetation that matters, but also key qualities such as accessibility, usability, vegetation diversity, and the presence of water bodies. Nevertheless, many epidemiologic studies still rely on single indicators such as the Normalized Difference Vegetation Index (NDVI), which—while useful—cannot capture the broader dimensions of nature beyond presence or absence (Jarvis et al., 2020; Donovan et al., 2022). High values of NDVI can be “green but unreachable,” and identical numeric shifts may denote very different vegetation cover depending on scale and species composition (AdlI and Labib, 2023). These limitations are especially important for policies that rely on single greenness metrics and may not reflect meaningful or equitable health benefits (Cities, 2021; Martin et al., 2025; Keeler et al., 2019). To ensure that greenness measurement can meaningfully inform policies with real-world public health impact, more nuanced exposure metrics and stronger evidence linking them to health outcomes are urgently needed. Moreover, urban environments consist of complex mixtures of interrelated natural features that rarely occur in isolation. Examining multiple dimensions of urban nature simultaneously is therefore essential for capturing the co-occurring and interacting environmental domains that shape daily exposure conditions—an idea central to the exposome framework. Such a multidimensional assessment also provides a clearer foundation for evidence-based policy and urban design.

Urban nature remains inequitably distributed across the U.S. (Klompmaker et al., 2023). These exposure gaps shape unequal burdens of environmental stressors and unequal access to protective resources, contributing to persistent disparities in cardiovascular disease. Equally important, vulnerability differs across populations: the same exposure to urban nature may yield different health benefits depending on underlying social, economic, and biological factors (Fernández Núñez et al., 2022; Núñez et al., 2022). Clarifying how urban nature promotes cardiovascular health across different population groups is also a critical step in informing targeted policy.

To address these gaps, we developed a multidimensional exposure classification of urban nature, incorporating high-resolution indicators of land cover, biodiversity, and park accessibility into empirically derived profiles that reflect real-world combinations of environmental conditions. Our primary aim is to understand the relationship between neighborhood urban nature profiles with ASCVD in New York City residents aged ≥ 55 years. We also assessed whether these associations differ by sex and race/ethnicity, as well as the neighborhood-level poverty and race/ethnicity.

## 2. Methods

### 2.1. Study Population, outcome, and covariates

This retrospective cohort study included New York City residents aged 55 years or older who received primary care at the Mount Sinai Health System (MSHS) between 2013 and 2022. Electronic health record (EHR) data were obtained from the Mount Sinai Data Warehouse. We identified ASCVD events using ICD-10 codes. We included ICD-10 codes for acute myocardial infarction (I21.), chronic ischemic coronary heart disease (I25.1, I25.7, I25.8), ischemic stroke (I63., I64, I65., I66.), peripheral arterial disease (I70.2, I73.9), and aortic atherosclerosis (I70.0) according to the 2019 ACC/AHA Guideline on the Primary Prevention of Cardiovascular Disease (Arnett et al., 2019)(Table S1). We recorded the earliest match occurring on or after the first-encounter year as the ASCVD date. Person-time began on January 1 of the first calendar year with a documented encounter and ended at the earliest of the following: ASCVD diagnosis, death, address change, two consecutive years without any documented encounter (censoring for loss to follow-up), or December 31, 2022. Demographic data, including age, sex, race/ethnicity, and insurance information, were obtained from electronic health records. Neighborhood socioeconomic context was linked by census tract and year to American Community Survey 5-year estimates, including percentages of residents under the federal poverty threshold, and identifying as non-Hispanic Black.

We excluded individuals who lacked valid covariate data (8 %), had no geocoded address during follow-up (1.4 %), or had a recorded ICD-10 diagnosis of ASCVD prior to 2013 (15.9 %) (Fig. S1). This study was approved by the Institutional Review Board at the Icahn School of Medicine at Mount Sinai (STUDY-22-01400), with a waiver of informed consent.

### 2.2. Exposure assessment and profile clustering

Residential addresses in MSHS EHRs are updated during clinical encounters when patients report an address change. We geocoded each participant’s address for every year and created 300-meter buffers around them. This spatial scale is commonly used in environmental epidemiology and is suitable for capturing neighborhood environments (Sadeh et al., 2021). We generated eight different urban nature exposure metrics using diverse environmental datasets available for New York City. Metrics were selected a priori to comprehensively capture influences on health outcomes across multiple dimensions: levels of engagement with nature as described by Pretty (2004), mechanisms outlined by Nieuwenhuijsen et al. (2017), and pathways defined by Markevych et al. (2017).

Exposures were calculated within the 300-meter buffer. We calculated: (1) The percentage of pixels classified as **blue space, tree canopy, and grass/shrub land cover,** using the light Detection and Ranging (LIDAR)-based six-inch, eight-category land cover raster dataset (University of Vermont Spatial Analysis Laboratory icwNYCOoTaINO and Geographics, 2017); (2) The **mean green view index (GVI)** to capture street-level vegetation coverage, using street view imagery from the Treepedia dataset (Li et al., 2015; Li, n.d); (3) The number of **street trees,** obtained from the New York City Street Tree Census (DPR) DoPaR., 2015), which includes all street trees in the city; (4) The **Shannon index**, a biodiversity metric estimating tree species proportions, was also derived from the Street Tree Census (DPR) DoPaR., 2015); (5) The total **number of parks** and (6) **large parks service areas**, obtained from a spatial dataset created by Spangler et al. (2023), which includes all parks and gardens across the continental United States. The latter represents 10-minute walking service areas around large parks (defined as ≥ 10 ha). Due to limited data availability, each exposure was calculated at a single time point between 2013 and 2022 and imputed for the remaining years. Details on operational definitions, data sources, spatial resolution, and year of measurement are provided in Table S2.

We applied min–max normalization to scale the eight environmental metrics and used k-means clustering to generate urban nature exposure profiles. K-means allows us to generate data-driven profiles without imposing subjective weighting or collapsing variables into a composite index. The optimal number of clusters was determined using the elbow method based on a Scree plot of within-cluster sum of squares. The resulting profiles reflect the environmental combinations that empirically co-occur across New York City, rather than all theoretically possible permutations of natural features. Clusters were assigned descriptive names based on the dominant environmental characteristics present in each profile to facilitate interpretation (e.g., high tree cover, water access, or proximity to parks) (Carroll et al., 2020).

**Fig. 1.**
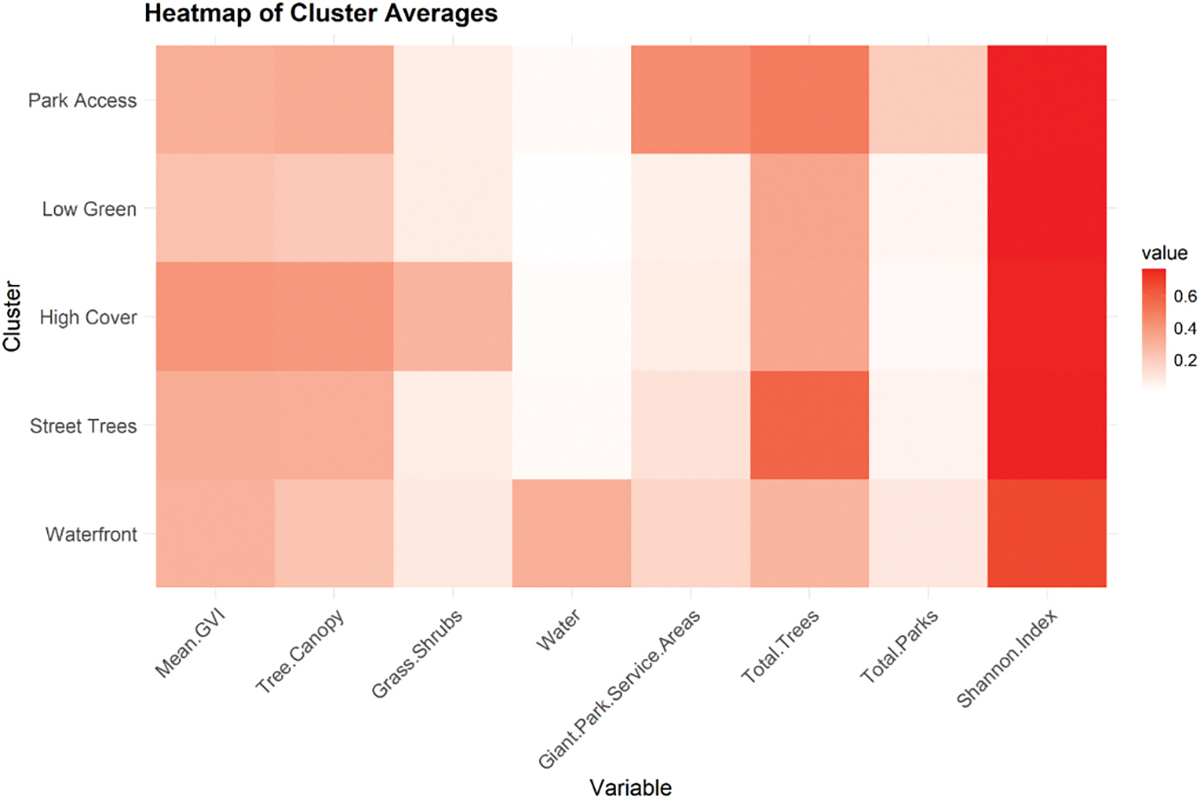
Heatmap of scaled average values of eight environmental metrics across the five urban nature exposure profiles. Each cell represents the normalized mean of a given environmental feature within a cluster, with darker colors indicating higher relative values. Profiles were defined using k-means clustering on min–max normalized input variables. Profiles were named after the dominant environmental features in each cluster.

**Fig. 2.**
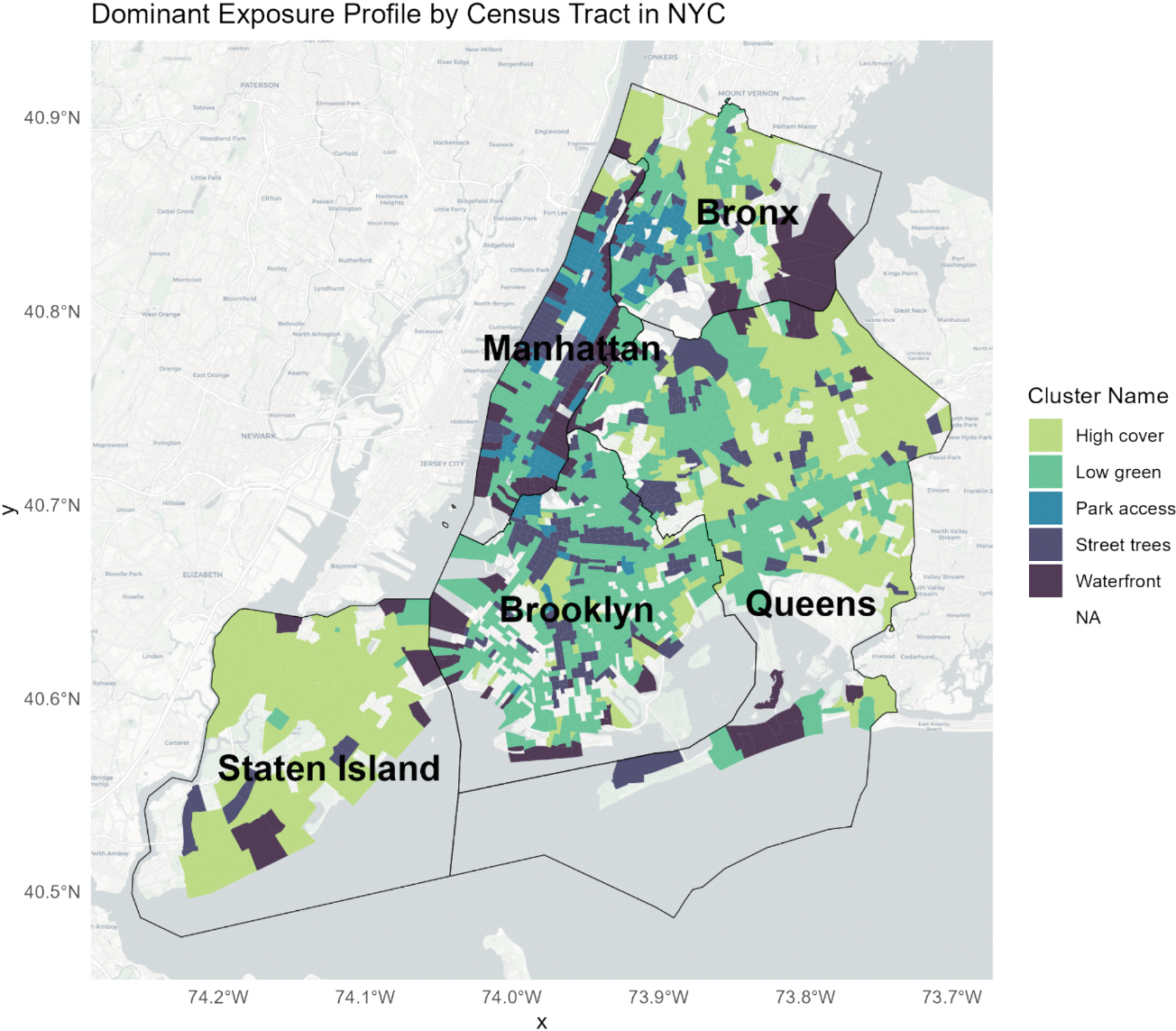
Spatial distribution of the dominant exposure profile by census tract across New York City. Each tract is shaded according to the modal cluster assignment among participants. Tracts with no observations are left transparent. Borough borders are overlaid and labeled in black.

We identified five profiles representing distinct urban nature profiles and named them according to their dominant feature (Fig. 1). The *Low green* profile, reflecting the lowest levels of urban nature, had the lowest mean GVI (11.1), tree canopy coverage (14.9 %), and blue space (0.4 %). It also had the fewest parks (1.97) and park service areas (2.2) on average. The *Street Trees* profile presented the highest number of street trees (512.7) and a high Shannon index (2.77), with moderate canopy (21.5 %) and low levels of blue space (1.5 %) and grass/shrub cover (4.3 %). The *High cover* profile exhibited high canopy (26.6 %), grass/shrub presence (15.7 %), and GVI (18.6, SD 5.0), but relatively limited access to large parks (1.65), service areas (2.67) and blue space (1.04). The *Park access* profile combined moderate-to-high tree canopy (22.5 %) with the greatest average access to parks (5.9) and park service areas (15.6). It had a high street tree count (439.5) and low grass/shrub cover (4.2 %). Finally, the *Waterfront* profile featured high blue space exposure (21.3 %) along with low levels of tree canopy (16.2 %), grass/shrub cover (5.1 %), and street trees (255.4), and more modest park access (3.3) and park service areas (5.8) on average. The spatial distribution of profiles shows coherence across the city without aligning strictly to borough boundaries, suggesting that the clustering approach captured meaningful ecological variation independent of administrative divisions. (Fig. 2).

### 2.3. Statistical analysis

Baseline characteristics were summarized overall and stratified by sex, race, and urban-nature profiles. We estimated the association between environmental exposure profiles and ASCVD events using Cox proportional hazards models, with the Low-green profile as the reference and death treated as a competing risk. The primary model included cluster membership as the main exposure and was adjusted for race/ethnicity (collapsed into White vs. Non-White), insurance type (Medicaid, Medicare, Private Insurance, Self-Pay, or Other), age, sex, and census tract-level covariates, including percent below the poverty line and percent non-Hispanic Black residents. We selected these covariates *a priori* based on their relevance as potential confounders of the relationship between neighborhood environments and ASCVD.

As a sensitivity analysis, we additionally adjusted for the three domains of the CDC Environmental Justice Index (EJI)—social vulnerability, environmental burden, and health vulnerability. These domains capture a broad set of neighborhood and built-environment characteristics at the census tract level. In these models, the EJI domain scores were included in place of the two neighborhood-level covariates used in the main analysis to evaluate the robustness of our findings to alternative measures of neighborhood context.

As a secondary analysis, we assessed whether the association between environmental profiles and ASCVD events varied across key sociodemographic and neighborhood strata. To test for effect modification, we added interaction terms between cluster membership and each of the following variables: sex, race/ethnicity, % poverty, and % non-Hispanic Black. For categorical modifiers (sex and race/ethnicity), we estimated hazard ratios for each environmental cluster within strata of the modifier and compared them using pairwise contrasts. For continuous modifiers (% poverty and % non-Hispanic Black), we estimated cluster-specific hazard ratios at the 25th and 75th percentiles of each variable and conducted contrasts at each level. All results are reported as hazard ratios (HRs) with 95 % confidence intervals (CIs). All analyses were conducted in R version 4.3.

## 3. Results

The final analytic sample included 36,830 individuals, contributing a total of 139,253 person-years. The mean age was 69.4 years (SD: 8.3); 64.8 % were female, 58.4 % identified as Non-White, and 4.2 % were covered by Medicaid. On average, participants resided in neighborhoods where 17.9 % of residents lived below the federal poverty line, and 16.3 % were non-Hispanic Black. At the cluster level, 31.1 % lived in *Low-green* areas, followed by 25.7 % in *Street trees*, 23.2 % in *Park access*, 11.0 % in *High cover*, and 9.0 % in *Waterfront* profiles. Over the follow-up period, 28.7 % of individuals experienced ASCVD (Table 1). Table S3 provides participant characteristics stratified by clusters.

Compared with the Low-green profile, the Waterfront, Park Access, and Street Trees profiles were each associated with lower ASCVD risk in fully adjusted Cox models (Table 2). Hazard ratios were 0.88 (95 % CI: 0.81–0.95) for Waterfront, 0.89 (95 % CI: 0.84–0.94) for Park Access, 0.92 (95 % CI: 0.87–0.98) for Street Trees, and 0.95 (95 % CI: 0.88–1.02) for High Cover. In the sensitivity analyses adjusting for the three CDC-EJI domains (social vulnerability, environmental burden, and health vulnerability), the direction and relative magnitude of associations across profiles were generally consistent with the main results, with only modest differences in effect sizes and precision (Table S4).

**Fig. 3.**
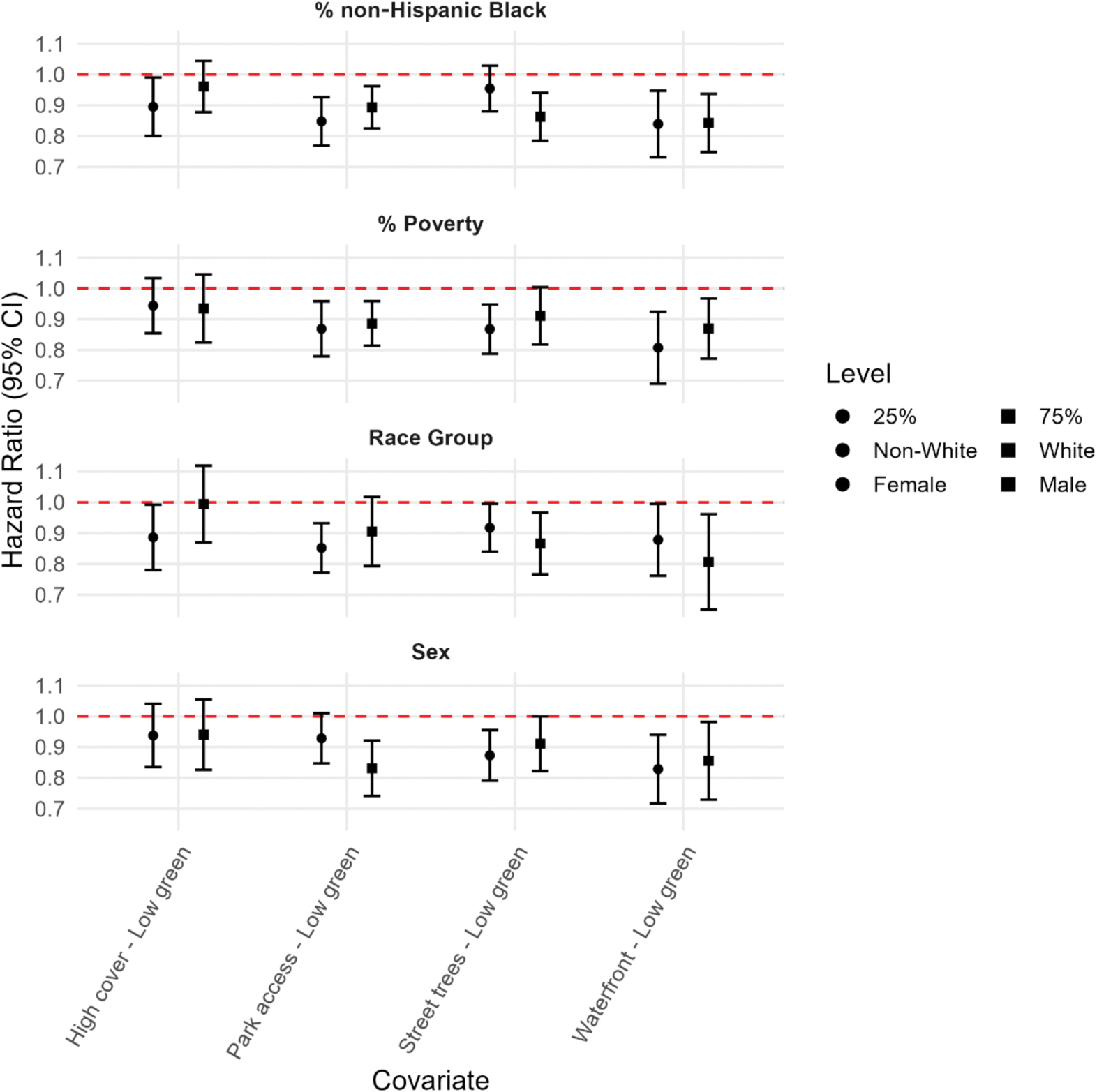
Hazard ratios (HRs) and 95% confidence intervals (CIs) comparing Nature Profiles across strata of sex, race/ethnicity, neighborhood poverty (%), and percent non-Hispanic Black residents. Results are from Cox proportional hazards models, including interaction terms between cluster membership and each modifier and death as a competing risk.

The secondary analysis highlighted several subgroup differences that complement the primary findings (Fig. 3, Table S5). A significant interaction term indicated that the association between the Park Access profile and ASCVD risk differed by sex, with stronger protective associations observed for males (interaction p-value: 0.04). Similarly, Street Trees were more protective in areas with higher percentages of non-Hispanic Black residents (interaction p-value: 0.02).

## 4. Discussion

In this EHR-based cohort of New York City residents aged 55 years or older, living in urban nature-rich environments was associated with a lower risk of ASCVD relative to Low Green. However, neighborhoods classified as High Cover where not substantially protective. The protective role of Park Access was modified by sex, and the protective role of Street Trees was modified in areas with higher percentages of non-Hispanic Black residents.

Our main results align with existing literature: nature-rich profiles are protective against ASCVD. Several conceptual frameworks have attempted to organize the diverse ways through which nature benefits human health. Pretty (2004) proposed a hierarchy of human engagement with nature comprising three levels that elicit distinct psychological and physiological responses. The first level involving incidental sensory exposure was initially limited to visual contact, such as viewing nature through a window, but now is understood to include other senses as well, as hearing birdsong. The second level refers to direct incidental exposure while engaged in unrelated activities, such as commuting through parks or reading in a garden. The third and most immersive level involves direct interaction with nature through intentional activities like gardening or trekking (Pretty, 2004). Nieuwenhuijsen et al. (2017) broadly categorized six mechanisms through which urban nature exerts health benefits, including environmental exposure reduction (e.g., air pollution, heat), promotion of physical activity, facilitation of social cohesion, enhancement of biodiversity, the role of biogenic emissions (e.g. phytoncides from trees), and the potential for restorative effects (e.g. stress reduction and emotional recovery). Markevych et al. (2017) proposed three interwoven pathways through which nature supports health: mitigation, restoration, and instoration. The mitigation pathway refers to nature’s role in reducing exposure to environmental stressors such as air pollution, noise, and excess heat. The restoration pathway involves the recovery of depleted resources, including stress reduction and attention restoration. Finally, the instoration pathway reflects nature’s capacity to build new resources, such as promoting physical activity, fostering social cohesion, and supporting psychological resilience(Markevych et al., 2017).

Moreover, our results also support the idea that increased nature cover, while potentially relevant, might not be the main driver of the urban nature benefits on cardiovascular health. The High cover profile captures overall vegetation density, reflecting environments rich in natural coverage but potentially lacking in physical or social accessibility. This profile aligns with much of the existing literature on urban greenness and health, which often relies on satellite-based indices like NDVI to quantify exposure. A recent systematic review of 63 studies across 21 locations found consistent evidence that higher levels of greenness were inversely associated with cardiovascular risk factors and major events, including hypertension, ischemic heart disease, and CVD mortality (Lai et al., 2023). Another review focusing on older adults’ wellbeing found a strong relationship between urban green space features and subjective wellbeing (Xu et al., 2022). However, a study found that perceived access to greenness had greater protective associations against cardiometabolic risk factors than NDVI, tree cover, and overall vegetation cover (Knobel et al., 2021). Another study found that greenspace quality played a greater protective role than green cover in reducing the risk of cardiometabolic disease (Liu et al., 2023).

The Park Access profile captures proximity and accessibility to natural areas, which may influence health through recreational activity, social interaction, and restorative experiences. Evidence shows that public parks provide physical, psychological, and social benefits to urban residents. For example, a study of 44 U.S. cities found that greater park coverage was one of the strongest predictors of self-reported wellbeing(Larson et al., 2016). Another study in Beijing found that better spatial accessibility to parks—especially at the regional scale—was associated with lower anxiety and depression symptoms among older adults, highlighting the importance of walkable access to parks (Zhao et al., 2023).

The Waterfront profile, despite featuring lower vegetation levels, was consistently associated with reduced ASCVD risk, potentially reflecting the distinct psychological and physiological benefits of blue space exposure. A narrative review proposed a conceptual framework outlining how aquatic environments promote well-being through both direct exposure and feedback loops tied to nature engagement(White et al., 2020). A systematic review of blue care interventions found consistent evidence for mental health benefits and increased social connectedness(Britton et al., 2020). Similarly, a review focused on older adults highlighted blue space’s therapeutic potential, with proximity and sensory richness linked to better mental health(Guan et al., 2025).

The Street Trees profile, characterized by high street-level vegetation density and diversity, was also associated with reduced ASCVD risk. A conceptual review highlighted the importance of evaluating street trees within an urban ecosystem services framework, noting their multifaceted contributions to climate regulation, air quality, and aesthetic value (Salmond et al., 2016). A large-scale study in China found that greater exposure to street greenery was associated with lower prevalence of cardiovascular disease(Wang et al., 2022). Another study in California showed that greater biodiversity of street trees, measured by the Shannon Index, was linked to lower mortality from heart disease and stroke (Giacinto et al., 2021).

Our secondary findings reinforce prior evidence that sociodemographic factors can modify the cardiovascular benefits of urban nature. Prior reviews have reported sex-specific patterns, with women benefiting more from greenness for some cardiovascular outcomes and men for others(Fernández Núñez et al., 2022). Other research documenting socioeconomic status and racial disparities in access to and quality of green space suggests that more advantaged communities may disproportionately benefit from urban greening initiatives (Browning and Rigolon, 2018).

This study leverages a large, diverse cohort drawn from a real-world clinical population, enhancing the generalizability of findings within an urban context. ASCVD outcomes were identified through EHRs, providing clinically verified diagnoses that reduce the risk of recall bias and misclassification. Environmental exposures were assessed at the individual level using 300-meter residential buffers. This distance was selected a priori based on extensive evidence in environmental epidemiology indicating that a 300-meter radius approximates an individual’s immediate neighborhood environment and captures features that are regularly seen, accessed, or traversed in daily life. By incorporating a multidimensional characterization of urban nature—rather than relying solely on single greenness metrics such as NDVI—our analysis captures a more ecologically and socially grounded view of environmental influences on cardiovascular health.

This study incorporated eight distinct urban-nature metrics representing vegetation cover, biodiversity, street-level greenery, blue space, and park accessibility. These measures were selected because they are openly available, interpretable, and feasible to implement in various population-based studies using large EHR cohorts. Although additional approaches such as LiDAR-derived canopy volume(Donovan et al., 2019), survey-based measures of perceived or subjective access to nature(Hart et al., 2022), and landscape-ecology indicators (Chi et al., 2025) are increasingly available, they were not incorporated in this analysis because the focus of this work was on exposure metrics that are broadly accessible and easily reproducible. These more advanced methods represent valuable opportunities for future studies to further refine multidimensional assessments of urban nature.

This study has several limitations. First, urban nature exposures were measured cross-sectionally and do not capture temporal variation. While this limits our ability to assess cumulative or time-varying effects, features like green infrastructure and park access tend to change slowly over time, especially relative to short-term exposures such as air pollution or temperature. As such, a cross-sectional snapshot still offers meaningful insight into environmental patterns relevant for long-term planning, especially for urban nature exposures that have limited year-to-year variation. Second, although we adjusted for a range of individual- and neighborhood-level sociodemographic covariates, residual confounding from unmeasured behavioral, psychosocial, or environmental factors likely remains. The inclusion of detailed electronic health records and neighborhood data helps reduce—but cannot eliminate—such confounding, so our findings should be interpreted with appropriate caution. Third, while our results are drawn from New York City—a unique urban setting with distinctive spatial and social structures—they offer valuable insight into how diverse urban nature configurations relate to cardiovascular health. Finally, because cluster sizes differed, comparisons across profiles should be interpreted with caution. Our interpretation emphasizes the overall pattern of effect sizes and confidence intervals—rather than statistical significance alone—to draw inferences about the relationship between multidimensional urban-nature environments and ASCVD risk.

## 5. Conclusion

Our findings contribute to the growing evidence that urban nature provides meaningful cardioprotective benefits. Importantly, diverse types of nature-rich environments beyond cover—whether through park access, tree canopy, or blue space—appear to confer similar protection relative to low-nature areas, though the effects may differ across population groups. From a policy perspective, these results highlight the importance of tailoring nature-based interventions beyond simply increasing greenness. Equitable urban design should consider multiple forms of accessible, biodiverse nature to maximize health benefits across diverse communities.

## Supplementary Material

1

## Figures and Tables

**Table 1:** Baseline Characteristics of Study Population (N = 36,830).

Variable	Value

Sex, female, N (%)	23,848 (64.8)
Race, N (%)	
Non-White	21,500 (58.4)
White	15,330 (41.6)
Insurance Type, N (%)	
Medicaid	1,546 (4.2)
Other	35,284 (95.8)
Age (Mean, SD)	69.44 (8.33)
Neighborhood Characteristics (Mean, SD)	
% Below Federal Poverty Line	17.90 (11.96)
% Non-Hispanic Black Residents	16.28 (21.01)
Nature Profile, N (%)	
Low green	11,456 (31.1)
Waterfront	3,324 (9.0)
Street trees	9,472 (25.7)
High cover	4,036 (11.0)
Park access	8,542 (23.2)
Events	
ASCVD cases	10,580 (28.7)
Deaths	815 (2.2)

**Table 2:** Hazard ratios (HRs) and 95 % confidence intervals (CIs) for the association between each urban nature exposure profile and ASCVD events with death as a competing risk.

Urban Nature Profile	Crude HR (95 % CI)	Adjusted HR (95 % CI)

Waterfront	0.86(0.80, 0.92)*	0.88(0.81, 0.95)*
Street trees	0.84(0.79, 0.88)*	0.92(0.87, 0.98)*
High cover	0.87(0.81, 0.93)*	0.95(0.88, 1.02)
Park access	0.96(0.91, 1.01)	0.89(0.84, 0.94)*

Estimates are from the primary Cox proportional hazards model using the Low-green profile as the reference group. The adjusted model was adjusted for race/ethnicity (White vs. Non-White), insurance type, age, sex, percent below the poverty line, and percent non-Hispanic Black residents. Asterisks (*) indicate statistically significant associations at p < 0.05.

## Data Availability

The data that has been used is confidential.

## Appendix A. Supplementary data

Supplementary data to this article can be found online at https://doi.org/10.1016/j.envint.2025.110033.
