# Supplementary material for: Beyond greenness: multidimensional urban nature profiles and arteriosclerotic cardiovascular risk
Source: Environ Int. Author manuscript; Available in PMC 2026 Feb 27. (PMC12947129; doi:10.1016/j.envint.2025.110033)
Supplement: 1 [file NIHMS2140430-supplement-1.docx]

**SUPPLEMENTARY MATERIALS**

**Table S1.** ICD-10 codes for Atherosclerotic Cardiovascular Diseases (ASCVD)

| **ASCVD Category** | **ICD-10 Code** | **Description** |
| --- | --- | --- |
| **Acute Myocardial Infarction** | I21.0 | ST elevation (STEMI) myocardial infarction of anterior wall |
|  | I21.1 | STEMI of inferior wall |
|  | I21.2 | STEMI of other sites |
|  | I21.3 | STEMI of unspecified site |
|  | I21.4 | Non–ST elevation (NSTEMI) myocardial infarction |
|  | I21.9 | Acute myocardial infarction, unspecified |
| **Chronic Ischemic Heart Disease / Coronary Atherosclerosis** | I25.10 | Atherosclerotic heart disease of native coronary artery without angina pectoris |
|  | I25.11 | Atherosclerotic heart disease of native coronary artery with angina pectoris (including I25.110–I25.119) |
|  | I25.7 | Atherosclerosis of coronary artery bypass graft(s) |
|  | I25.8 | Other forms of chronic ischemic heart disease |
| **Ischemic Cerebrovascular Disease** | I63.x | Cerebral infarction (all ischemic stroke subcodes) |
|  | I64 | Stroke, not specified as hemorrhage or infarction |
|  | I65.x | Occlusion and stenosis of precerebral arteries, not resulting in cerebral infarction |
|  | I66.x | Occlusion and stenosis of cerebral arteries, not resulting in cerebral infarction |
| **Peripheral Arterial Disease** | I70.2x | Atherosclerosis of arteries of extremities (all subcodes) |
|  | I73.9 | Peripheral vascular disease, unspecified |
| **Aortic Atherosclerosis** | I70.0 | Atherosclerosis of aorta |

**Figure S1.** Flowchart showing derivation of the final analytic sample. Starting from 46,746 eligible individuals, exclusions were applied for missing covariates, invalid addresses, and prior ASCVD diagnoses, resulting in a final sample of 36,830 participants.


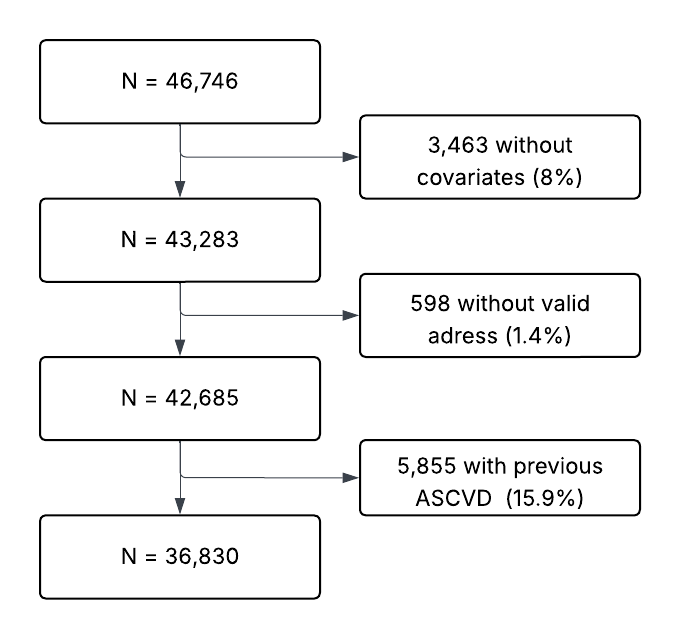


**Table S2.** Description of the eight urban-nature exposure metrics used to construct multidimensional environmental profiles. These metrics represent slowly changing structural features of the urban environment and are treated as time-stable measures of long-term neighborhood exposure.

| **Exposure Metric** | **Operational Definition** | **Data Source** | **Resolution** | **Year** |
| --- | --- | --- | --- | --- |
| **Blue Space (%)** | Percentage of land area within the 300-m buffer classified as water. | NYC LiDAR Land Cover Raster (OTI)^52^ | 6-inch raster | 2017 |
| **Tree Canopy (%)** | Percentage of land covered by tree canopy (>8 ft). |  |  |  |
| **Grass/Shrub Cover (%)** | Percentage of land covered by grass and shrubs (<8 ft). |  |  |  |
| **Total Trees** | Count of living street trees within the buffer. | NYC Street Tree Census (NYC DPR)^33^ | Point | 2015 |
| **Shannon Index** | Tree species diversity index (richness + evenness). |  |  |  |
| **Mean GVI (Green View Index)** | Average visible vegetation from Google Street View imagery using computer-vision algorithms. | MIT Treepedia (Google Street View–derived)^32^ | Point | 2019 |
| **Total Parks** | Number of parks intersecting the 300-m buffer. | Spangler et al. (2023)^34^ | Polygon | 2021 |
| **Giant Park Service Areas** | Count of 10-minute walking-service areas for parks ≥10 ha intersecting the buffer. |  |  |  |

**Table S3:** Baseline Characteristics of Study Population stratified by cluster type.

| **Variable** | Low Green  (N = 11,456) | Waterfront  (N = 3,324) | Street Trees  (N = 9,472) | High Cover  (N = 1,054) | Park Access  (N = 8.542) |
| --- | --- | --- | --- | --- | --- |
| Sex, female , N (%) | 7224 (63.1) | 2265 (68.1) | 6083 (64.2) | 2587 (64.1) | 5689 (66.6) |
| Race, N (%) |  |  |  |  |  |
| Non-White | 7070 (61.7) | 2029 (61.0) | 4022 (42.5) | 2041 (50.6) | 6338 (74.2) |
| White | 4386 (38.3) | 1295 (39.0) | 5450 (57.5) | 1995 (49.4) | 2204 (25.8) |
| Insurance Type, N (%) |  |  |  |  |  |
| Medicaid | 643 (5.6) | 94 (2.8) | 303 (3.2) | 170 (4.2) | 336 (3.9) |
| Other | 10813 (94.4) | 3230 (97.2) | 9169 (96.8) | 3866 (95.8) | 8206 (96.1) |
| Age (Mean, SD) | 69.35 (8.16) | 69.37 (8.28) | 69.69 (8.32) | 69.41 (8.30) | 69.33 (8.60) |
| Neighborhood Characteristics (Mean, SD) |  |  |  |  |  |
| % Below Federal Poverty Line | 17.32 (10.27) | 22.38 (15.16) | 11.83 (8.05) | 13.33 (10.19) | 25.47 (12.05) |
| % Non-Hispanic Black Residents | 13.52 (21.59) | 21.62 (21.73) | 7.92 (13.95) | 15.89 (22.96) | 26.86 (20.43) |
| Events |  |  |  |  |  |
| ASCVD cases | 3337 (29.1) | 929 (27.9) | 2507 (26.5) | 1054 (26.1) | 2753 (32.2) |
| Deaths | 227 (2.0) | 69 (2.1) | 167 (1.8) | 65 (1.6) | 287 (3.4) |

**Table S4.** Hazard ratios (HRs) and 95% confidence intervals (CIs) for the association between each urban nature exposure profile and ASCVD events with death as a competing risk. Estimates are from the sensitivity Cox proportional hazards model using the Low-green profile as the reference group. Adjusted model was adjusted for race/ethnicity (White vs. Non-White), insurance type, age, sex, social vulnerability, environmental burden, and health vulnerability. Asterisks (*) indicate statistically significant associations at p < 0.05.

| Urban Nature Profile | Adjusted HR (95% CI) |
| --- | --- |
| Waterfront | 0.72(0.53, 0.97)* |
| Street trees | 0.82(0.66, 1.02) |
| High cover | 0.96(0.71, 1.29) |
| Park access | 0.94(0.77, 1.14) |

**Table S5:** Hazard ratios (HRs) and 95% confidence intervals (CIs) comparing Nature Profiles across strata of sex, race/ethnicity, neighborhood poverty (%), and percent non-Hispanic Black residents. Results are from Cox proportional hazards models including interaction terms between cluster membership and each modifier, plus adjustment by insurance and age. Asterisks (*) indicate statistically significant contrasts at p < 0.05.

|  | Sex | | Race group | | % Poverty | | % NH Black | |
| --- | --- | --- | --- | --- | --- | --- | --- | --- |
| Contrast | **Female** | **Male** | **Non-White** | **White** | **25%** | **75%** | **25%** | **75%** |
| Waterfront / Low Green | 0.828 (0.741; 0.926)* | 0.855 (0.754; 0.97) | 0.878 (0.782; 0.987) | 0.807 (0.691; 0.942) | 0.807 (0.718; 0.907)* | 0.87 (0.789; 0.959)* | 0.839 (0.753; 0.935)* | 0.843 (0.767; 0.926)* |
| Street Trees / Low Green | 0.873 (0.804; 0.948)* | 0.911 (0.833; 0.995) | 0.918 (0.849; 0.991) | 0.867 (0.784; 0.958)* | 0.868 (0.801; 0.941)* | 0.911 (0.83; 0.999) | 0.955 (0.887; 1.028) | 0.863 (0.798; 0.933)* |
| High Cover / Low Green | 0.938 (0.846; 1.039) | 0.94 (0.839; 1.054) | 0.886 (0.797; 0.985) | 0.994 (0.878; 1.127) | 0.944 (0.863; 1.032) | 0.935 (0.837; 1.044) | 0.895 (0.814; 0.984) | 0.961 (0.884; 1.044) |
| Park Access / Low Green | 0.928 (0.856; 1.007) | 0.831 (0.76; 0.909)* | 0.852 (0.786; 0.923)* | 0.905 (0.809; 1.013) | 0.869 (0.795; 0.95)* | 0.886 (0.824; 0.952)* | 0.848 (0.784; 0.917)* | 0.893 (0.834; 0.957)* |
